# Radiocesium Transfer in Forest Insect Communities after the Fukushima Dai-ichi Nuclear Power Plant Accident

**DOI:** 10.1371/journal.pone.0171133

**Published:** 2017-01-26

**Authors:** Yumiko Ishii, Seiji Hayashi, Noriko Takamura

**Affiliations:** 1 Environmental Impact Assessment Section, Fukushima Branch, National Institute for Environmental Studies, Miharu Town, Tamura County, Fukushima, Japan; 2 Center for Environmental Biology and Ecosystem Studies, National Institute for Environmental Studies, Tsukuba, Ibaraki, Japan; University of Hyogo, JAPAN

## Abstract

To understand radiocesium transfer in the forest insect food web, we investigated the activity concentrations of radiocesium in forest insects in the Fukushima and Ibaraki Prefectures approximately 1.5–2.5 years after the Fukushima Dai-ichi Nuclear Power Plant. We analyzed 34 species of insects sampled from 4 orders and 4 feeding functional groups (herbivore, carnivore, omnivore, and detritivore) from three sites in each prefecture. ^137^Cs activity concentrations were lowest in herbivorous species and were especially high in detritivorous and omnivorous species that feed on forest litter and fungi. Radiocesium activity concentrations in any given species reflected the degree of contamination of that species’ primary food sources since radiocesium activity concentrations were found to be the lowest in leaves and grass and the highest in litter, bark, and fungi. This study confirmed that litter and other highly contaminated forest components such as fungi, decaying wood, bryophytes, and lichens serve as sources of ^137^Cs transfer into the forest insect community.

## Introduction

The forest ecosystems of Fukushima and its adjacent prefectures were severely contaminated with radionuclides after the Fukushima Dai-ichi Nuclear Power Plant (FDNPP) accident on 11 March 2011 [1,2]. For decades to come, the most biologically important radionuclide will be radiocesium because of its long half-life (30.1 years for ^137^Cs, 2.1 years for ^134^Cs) [2,3]. Many studies have reported that most of the Chernobyl radiocesium fallout still resides in surface layers in bioavailable form and continues to be a major potential source for transfer into living organisms even several decades after deposition [4,5]. Similarly, in Fukushima, radiocesium is expected to remain in the litter and upper soil layers of the forest floor for the long term [3].

Entry of radiocesium into forest ecosystems can potentially occur through two different pathways: the plant-based food chain and the detritus-based food chain. In the plant-based food chain, radiocesium in living plants moves into grazing herbivores and then into carnivores. In the detritus-based food chain, radiocesium enters the ecosystem via organisms feeding on litter and detritus (detritivores) and carnivores. Previous studies have reported highly contaminated litter to be the primary source of radiocesium in the forest ecosystem [6–8]. A survey of the forest invertebrate communities in the United Kingdom found the highest activity concentrations of ^137^Cs in invertebrate detritivores, such as earthworms (Oligochaetes) and woodlice (Isopoda) [8]. By broadly sampling organisms in forests and adjacent streams, including both vertebrates and invertebrates (fish, amphibian, reptile, arthropod, earthworms, etc.), Murakami et al. [6] found that detritivores are more contaminated with ^137^Cs than herbivores and carnivores at Fukushima.

The forest insect community constitutes a major route of radiocesium transfer to higher trophic organisms such as small mammals and birds. The highly varied feeding habits, life histories, and habitats of insects permit investigation of how radiocesium transfer from forest components into higher organisms occurs. There is particular concern for the effects of radiation on wildlife, including insects, as a result of the FDNPP accident [9–11]. However, compared with the numerous studies examining radioactive contamination of organisms used for human consumption, such as fish and game, only a few studies have been published about radionuclide accumulation in insects after the accident [12]. Previous studies conducted in European countries have reported on radionuclide transfer and accumulation of insects and other invertebrates [8,13–17], but it remains unclear how ^137^Cs uptake occurs in the entire insect food web and in relation to insect feeding habits.

In this study, we focused on radiocesium transfer in insect communities by investigating ^137^Cs activity concentrationsg in forest insects in the Fukushima and Ibaraki Prefectures over a period of 1.5–2.5 years after the Fukushima Dai-ichi reactor accident. To assess the distribution of radiocesium across insect communities and the influence of feeding ecology on radiocesium uptake, we collected insect samples from a wide range of insect species: We sampled species from four taxonomic orders (Coleoptera, Hemiptera, Lepidoptera, Orthoptera) and four feeding functional groups within those orders (herbivore, omnivore, carnivore, detritivore). To assess ^137^Cs uptake across different levels of contamination, we collected samples from a high-contamination area in Fukushima and from a low-contamination area in Ibaraki (^137^Cs deposition was 130–270 kBq m^−2^ at the Fukushima area and 13 kBq m^−2^ at the Ibaraki area according to the 4^th^ Airborne Radiation Monitoring by Ministry of Education, Culture, Sports, Science and Technology in 2011) [18]. To assess the distribution of radiocesium in insect food sources, we also sampled forest floor litter, tree leaves, grasses, bark, fungi, and bryophytes in both study areas.

## Materials and Methods

### Study area and sampling locations

**Fig 1:**
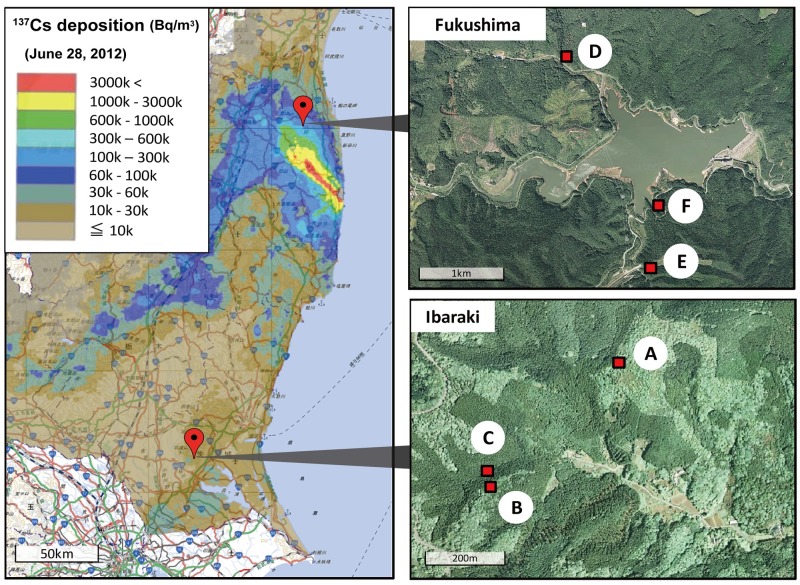
Locations of Study Sites in the Fukushima and Ibaraki Prefectures. Left panel shows ^137^Cs deposition (Bq m^−2^) in eastern Japan (The map was generated from the Distribution Map of Radiation Dose by MEXT, Japan, http://ramap.jaea.go.jp/map/). Right panels are the aerial photographs provided by the Geographical Survey Institute (http://maps.gsi.go.jp/development/ichiran.html), with red squares showing locations of study sites in the Ibaraki Prefecture (A, B, C) and Fukushima Prefecture (D, E, F).

Fig 1 shows the locations of the two forest study areas in the Ibaraki Prefecture (sites A–C) and in the Fukushima Prefecture (sites D–F). The Ibaraki study area was located approximately 160 km southwest of the FDNPP, close to Mt. Tsukuba. Two sites were located in secondary forest dominated by deciduous trees (sites A, B) and the third was located in a Japanese cedar (*Cryptomeria japonica*) plantation (site C). The Fukushima study sites were located 39 km northwest of the FDNPP, near Lake Udagawa. Sites D and F were secondary forest dominated by deciduous trees. Site E was a small open area surrounded by both deciduous and Japanese cedar forests. Light traps were used in the open area, and pitfall traps were set on the floor of the Japanese cedar forest. These traps are described in greater detail below. Permission was granted for the field study by the Kanto regional forest office and the Tohoku regional forest office of the Forest Agency.

### Sampling and specimen processing

Insect sampling was conducted during the summers of 2012 and 2013. Pitfall traps were used for ground-dwelling beetles, and light traps were used for moths and other flying insects. Grasshoppers were collected using a sweep net. For pitfall traps, circular 180-mL plastic drinking cups (7.5-cm diameter) containing acetic acid as bait were used. For each site, 60–150 traps were placed on the forest floor, and sampling took place for 2–28 days from June to September (see Table 1 for collection dates). Light trapping was conducted in the open area of site E in the Fukushima study area. For light traps, a white sheet (1.5 × 1.5 m) was hung behind two light sources (160-W mercury vapor and 20-W fluorescent UV), and insects were captured by hand. Light trapping was conducted for approximately 2 h between 18:00–21:00 h. Light trapping was conducted only at the Fukushima sites because we were unable to collect sufficient biomass for analysis in the Ibaraki sites because of the low ^137^Cs activity concentrations in samples in the preliminary test results. Sweep net sampling was conducted in an open area close to site D in 2013.

**Table 1:** Details of study Site. ^137^Cs activity concentrations and counting errors in Litter Samples are shown for each study site.

Prefecture	Study sites	Location (latitude, longitude)	Forest type	Insect collection	^137^Cs in litter (Bq kg^-^1, dry) in year 2012 and 2013
Ibaraki	A	(36.2012, 140.1359)	Deciduous forest	Pitfall traps (21 June–25 July, 2012)	736 ± 23 (2012)
				Pitfall traps (16–19 July, 2013)	248 ± 9 (2013)
	B	(36.1976, 140.1314)	Deciduous forest	Pitfall traps (21 June–25 July, 2012)	987 ± 24 (2012)
				Pitfall traps (16–19 July, 2013)	778 ± 22 (2013)
	C	(36.1980, 140.1312)	Cedar forest	Pitfall traps (21 June–25 July, 2012)	3435 ± 105 (2012)
				Pitfall traps (16–19 July, 2013)	1498 ± 39 (2013)
Fukushima	D	(37.8031, 140.7581)	Deciduous forest	Pitfall traps (8–19 Sep, 2012) Pitfall traps (17–18 July/9–18 Sep, 2013)	11354 ± 83 (2012)
					4980 ± 150 (2013)
	E	(37.7892, 140.7642)	Cedar forest Deciduous forest	Pitfall traps (8–19 Sep, 2012) Light trap (8 Sep, 2012)	34409 ± 158 (2012)
				Pitfall traps (17–18 July and 9–10 Sep, 2013) Light trap (9 Sep, 2013)	17359 ± 94 (2013)
	F	(37.7933, 140.7651)	Deciduous forest	Pitfall traps (8–19 Sep, 2012)	14145 ± 120 (2012)
				Pitfall traps (17–18 July and 9–10 Sep, 2013)	31099 ± 380 (2013)

Insect samples were sorted and identified to the species level. Several samples were identified only to the family level because of difficulties in species identification and because insufficient biomass was available for ^137^Cs determination if sorted to the species level. After measurement of fresh weight, samples were dried for >48 h at 60°C, then dry weights were determined. Individual samples of each species were combined and homogenized intact using a food processor. ^137^Cs concentration is reported for dry weight rather than wet because samples collected from pitfall traps vary greatly in fresh weight because of variations in acetic acid absorption.

Forest components, such as litter, tree leaves, grasses, barks, fungi, bryophytes were also sampled in 2012. Because fungi and bryophytes show a sporadic distribution, we sampled them only in the Fukushima sites in 2012. Leaf and grass samples were washed with water, dried at 70°C, weighed, and powdered using a food processor (see [19] for details). Litter, bark, fungi, and bryophytes were not washed but were dried and powdered similarly to leaves and grass.

### Radiocesium measurements

All samples were stored in plastic containers (U8 container, diameter = 50 mm, height = 62 mm). ^137^Cs activity concentrations were measured using germanium coaxial detectors (GC2518Canberra Japan, Tokyo, Japan; SEG-EMS GEM 35–70, Seiko EG&G Co. Ltd., Tokyo, Japan). Most samples were measured for <10% of the error counts per net area counts, and samples containing only a few becquerels of activity were measured for <15% of the error counts per net area counts. Standardized sources for calibrating the detectors were MX033U8PP (Japan Radioisotope Association, Tokyo, Japan) and EG-ML (Eckert & Ziegler Isotope Products, Valencia, CA, USA). The software Gamma Studio (SEIKO EG&G, Tokyo, Japan) was used to analyze γ-ray spectra. Activities of samples were corrected for radioactive decay to the date of sample collection and were expressed as Bq/kg. S1 and S2 Tables show the ^137^Cs activity concentrations (Bq kg^−1^ dry weight) in sampled insect species and forest components, respectively.

### Data analysis

After excluding values that fell below the detection limit of the instrumentation, data from 68 insect samples consisting of 34 species were used for the analysis of insect ^137^Cs activity concentrations. ^137^Cs activity concentrations tend to be lognormally distributed [20], so the ^137^Cs activity concentrations in insect and litter samples were log-transformed to fulfill requirements of normal distribution and homogeneity of variance. Whether ^137^Cs activity concentrations differed across feeding functional groups was assessed using a generalized linear mixed-effects model (GLMM). GLMM is an extension of the generalized linear model that takes into account both fixed and random effects [21]. In this study, the dependent variable was the ^137^Cs activity concentration in a given insect species. Fixed effects were functional feeding group, the ^137^Cs activity concentration in litter, sampling year (2012, 2013), and forest type (cedar vs. deciduous forest). Random effects were sampling site (A–F) and insect species. The functional feeding group to which a particular species was assigned (herbivore, carnivore, omnivore, detritivore) was based on its predominant food source. To determine the best model, we used likelihood ratio tests to compare the full model with nested models in which one of the predictor variables was omitted. If the omitted variable had no significant effect on the model, then that variable was removed from the model. This model was also selected as the best model using AIC (Akaike's Information Criterion) from models using all combinations of variables. Because we were interested in the differences between functional feeding groups, Tukey–Kramer post hoc tests were conducted to test multiple pairwise comparisons [22].

To compare the transfer of ^137^Cs into insects across different contamination levels in Fukushima and Ibaraki Prefecture, a concentration ratio (CR) was calculated for each species as Bq kg^−1^ dry weight of insect/Bq kg^−1^ dry weight of litter. Although different definitions of transfer have been developed for different purposes, we standardized ^137^Cs transfer into insect species to ^137^Cs activity concentrations in forest litter because litter is the most basal food resource in forest ecosystems.

All statistical analyses were performed with the software R, ver. 3.1.1 [23], using the optional package lme4 for GLMM analysis and the multcomp package for multiple comparisons.

## Results and Discussion

### ^137^Cs distribution in forest components

**Fig 2:**
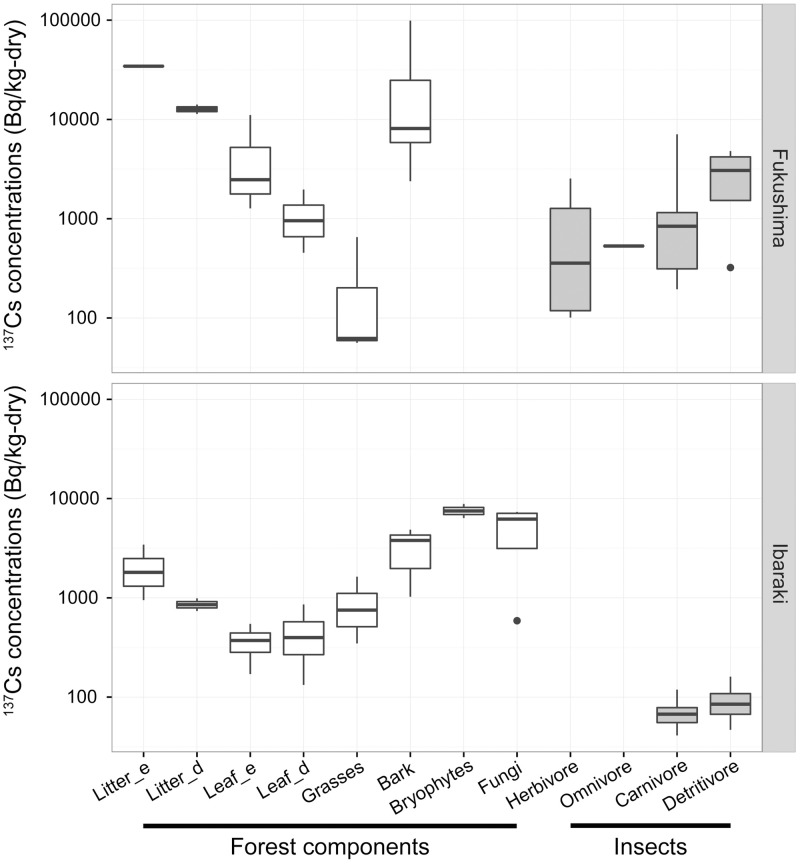
^137^Cs activity concentrations in Forest Components and Insects. ^137^Cs activity concentrations are shown for study sites in Fukushima (upper panel) and Ibaraki (lower panel) in 2012. Litter and leaf samples are shown separately for Japanese cedar forests (Litter_e and Leaf_e) and deciduous forests (Litter_d and Leaf_d). Dark horizontal lines represent the mean, with the box representing the 25th and 75th percentiles, the whiskers the 5th and 95th percentiles, and dots indicating outliers.

^137^Cs activity concentrations in the various forest components are shown in Fig 2. As expected, ^137^Cs mostly accumulated in the litter layer. Living leaves and grass had much lower ^137^Cs activity concentrations than litter in both study areas, and this was the case for both cedar and deciduous forests. ^137^Cs activity concentrations were higher in the litter and leaves of cedar forests than in those of deciduous forests. The reported higher ^137^Cs activity concentrations in evergreen species than in deciduous species have been attributed to the expansion of the foliar parts of the former, but not of those of the latter, at the time of fallout [24]. The finding that litter has a higher activity concentrations of ^137^Cs than leaves is also consistent with previous studies, which reported that most of the radioactive cesium deposited in Fukushima forests was rapidly transported to the forest floor within 1–2 years after deposition [25,26]. Although the samples from Fukushima had an order of magnitude higher activity of ^137^Cs than those from Ibaraki, the pattern of distribution of ^137^Cs among forest components was similar in both areas. The relatively lower levels in leaves and grass reflects a low rate of uptake from the soil by living plants.

In contrast to leaves and grass, bark, bryophytes, and fungi were highly contaminated. Previous investigators have found that fungi strongly accumulate radiocesium and play an important role in the uptake and retention of radiocesium in the organic layers of forest ecosystems [5,27]. Bryophytes and lichens are also known to passively accumulate high levels of radiocesium and retain radionuclides for long time periods because of their long life spans [28–30]. Thus, these forest components provide insect species not only a highly contaminated diet but also a contaminated habitat causing external radiation exposure.

The ^137^Cs activity concentrations in detritivorous insects were 1–2 orders of magnitude lower than the activity concentrations in litter in both the Ibaraki and Fukushima areas. Activity concentrations of ^137^Cs in herbivorous insects were similar to those in tree leaves and grass at Fukushima (no herbivorous insect samples were collected at Ibaraki.)

### Effect of insect feeding habit on ^137^Cs uptake

**Fig 3:**
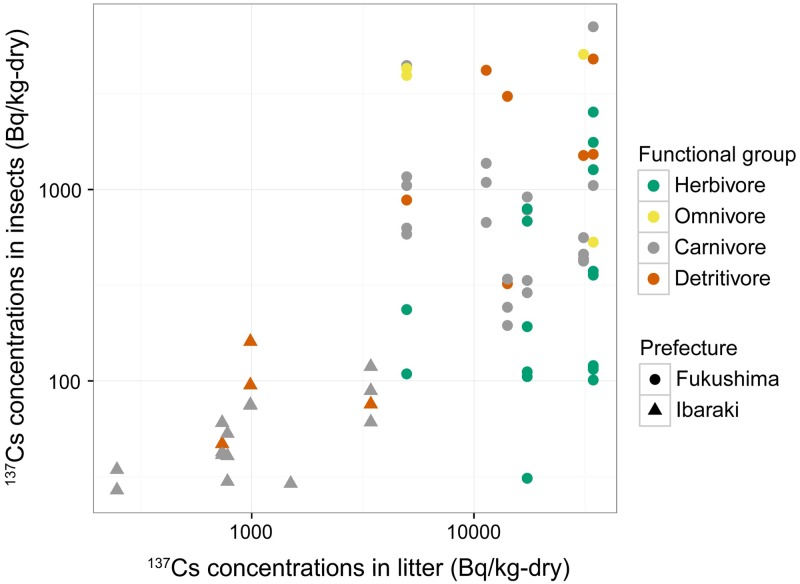
^137^Cs Activity Concentrations in Insect Feeding Functional Groups. ^137^Cs activity concentrations of insect species are shown in relation to the ^137^Cs activity concentrations in litter at the site. Colors indicate the functional feeding group to which species belongs: green, herbivore; yellow, omnivore; gray, carnivore; red, detritivore. Symbols indicate the sampling area: circle, Fukushima; triangle, Ibaraki.

Fig 3 presents a scatterplot of ^137^Cs activity concentrations in various insect species within the four functional feeding groups in relation to the ^137^Cs activity concentrations in litter contamination at the site where they were collected.

The GLMM analysis of the contribution of each variable to insect ^137^Cs contaminations indicated a significant effect of litter ^137^Cs activity concentration and functional feeding group, whereas no significant effect was found for both sampling year and forest type (Table 2). In the final model (Table 3), ^137^Cs activity concentrations in insect samples were positively correlated with those in litter (*P* < .001). Thus, the ^137^Cs activity concentrations in insects reflected the degree of contamination of litter at the study sites. ^137^Cs activity concentrations of litter are heterogeneous and are known to redistribute with time on the forest floor [31]. On average, ^137^Cs concentrations in both the litter and the ground beetle *Carabus albrecht* were lower in 2013 than in 2012 (Table 1, S2 Table). However, at site F, the ^137^Cs activity concentration of litter was higher in 2013 than in 2012 (Table 1). Although the mechanism is not entirely clear, the lateral transport of heterogeneously contaminated litter might have caused an increase in ^137^Cs activity concentrations because site F was located near the bottom of a hillslope [31]. In association with the increase in ^137^Cs activity concentration in the litter, ^137^Cs activity concentrations in *C*. *albrecht* also increased from 242.5 Bq kg^−1^ to 459.3 Bq kg^−1^ and 473.0 Bq kg^−1^ at site F (S2 Table). This confirms that ^137^Cs activity concentrations in insects reflected the degree of contamination of litter; therefore, CR values calculated from ^137^Cs activity concentrations in litter are appropriate to compare the transfer of ^137^Cs into insects despite heterogeneous distribution of ^137^Cs on the forest floor.

**Table 2:** Model selectionof GLMM for the ^137^Cs activity concentrations in insects. The effect of separately omitting each variable from the full model showing both AIC and chi-square test statistics.

Variable (omitted terms)	AIC	*P* (*χ*^2^)
Full model	177.4	
^137^Cs activity concentration in litter	188.9	**< 0.001**
Functional feeding group	184.9	**0.007**
Forest type	175.3	0.83
Sampling year	174.7	0.29

**Table 3:** The final model of GLMM for the ^137^Cs activity concentrations in insectsshowing estimates, standard errors, and *P*-values. Coefficients in bold indicate significant effects (*P* < .05).

Variable	Estimate	SE	t value	*P*
Intercept	–1.96	1.15	–1.69	0.10
^137^Cs activity concentration in litter	0.73	0.12	5.74	**<0.001**
Functional feeding group				
Herbivore—Omnivore	–1.85	0.74	–2.47	**0.05**
Herbivore—Carnivore	–1.16	0.42	–2.75	**0.02**
Herbivore—Detritivore	–1.83	0.55	–3.29	**0.004**
Ominvore—Carnivore	0.69	0.76	0.90	0.79
Ominvore—Detritivore	0.01	0.85	0.01	0.99
Carnivore—Detritivore	–0.67	0.57	–1.17	0.63

GLMM analysis also revealed the significant effect of functional feeding groups on insect ^137^Cs activity concentrations. Multiple comparison analysis showed that herbivores had significantly lower ^137^Cs activity concentrations than detritivores (*P* = .004), carnivores (*P* = .03), and omnivores (*P* = .05), but no significant differences were observed in its activity concentrations when the latter three functional groups were compared with one another (Table 3).

**Fig 4:**
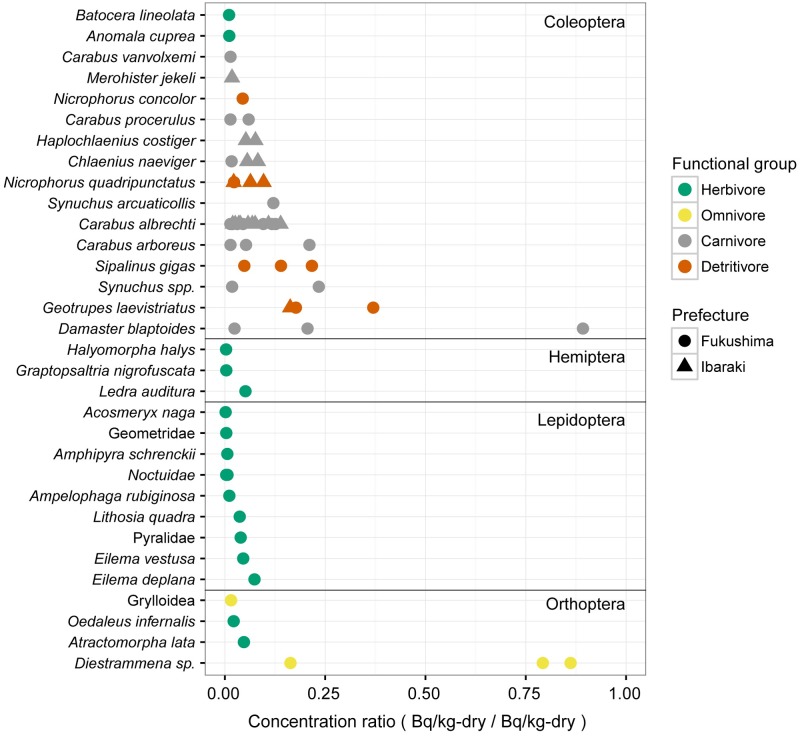
Concentration Ratio of ^137^Cs in Sampled Insect Species. CRs were calculated as Bq kg^−1^ insect dry weight/(Bq kg^−1^ litter dry weight. Species are grouped by the orders to which they belong (Coleoptera, Hemiptera, Lepdoptera, Orthoptera) with lines separating the orders. Colors indicate the functional feeding group to which species belongs: green, herbivore; yellow, omnivore, gray, carnivore; red, detritivore. Symbols indicate the sampling area: circle, Fukushima; triangle, Ibaraki.

Fig 4 shows the CR for each insect species/order collected. Values ranged from 0.003 to 0.89.

Although we have data on ^137^Cs activity concentrations for only carnivores and detritivores in Ibaraki sites, the CR values for the collected insect species were similar between Ibaraki and Fukushima sites. For example, CRs of the samples of *C*. *albrechti*, which were collected in large quantities at all sites, were similar between the Ibaraki and Fukushima sites (t-test, t = 0.01, df = 16, *P* = .99). These results suggest that uptake rate of ^137^Cs can be consistent regardless of amount of ^137^Cs depositions.

Comparing CRs across functional groups, herbivores showed the lowest values. Sampled herbivores included moths (Lepidoptera), herbivorous flying beetles (Coleoptera), stinging bugs (Hemiptera), and grasshoppers (Orthoptera). The low CRs for herbivorous insects reflect their diet of living plant tissues, which were found to contain relatively low activity concentrations of ^137^Cs compared to litter (Fig 2). The three herbivores with slightly higher CRs (*Lithosia quadra*, *Eilema deplana*, *E*. *vestusa*) are members lichen moth family Arctiidae. The CRs were likely higher because the larvae of these moth species feed on highly contaminated lichen and algae growing on trees or stones.

The CRs in carnivore species were overall higher than those in herbivores. Predominant carnivore species in the sample were ground-dwelling beetles of the family Carabidae. Ground beetles capture and consume a wide range of other soil-dwelling organisms, including detritivorous invertebrates and earthworms.

The higher CR values for carnivore species relative to herbivore species reflect the high contamination levels of the organisms in their diets. We did not investigate ^137^Cs activity concentrations in earthworms and soil invertebrates, but earthworms and detritivorous soil invertebrates such as springtails (Collembola) and woodlice (Isopoda) were consistently found to have higher ^137^Cs activity concentrations than other invertebrate groups [8,15]. Copplestone et al. [15] also standardized the activity concentration of ^137^Cs in living organisms to those in litter, reported ratios of 0.9–1.33 for earthworms showing relatively high CR value compared with carnivores in Fig 4. The snail-feeding Carabidae beetle, *Damaster blaptoides*, had the highest CR of all carnivore species collected, which also likely resulted from the contamination of terrestrial snails. Some species of terrestrial snails whose diet contain algae, lichens and fungi have been reported to accumulate relatively high amount of radiocesium than other herbivorous species [8,32].

Among detritivores and omnivores, high CR values were found for species that feed on fungi or litter, and relatively low values were found for carrion feeders. In this study, four species of Coleopteran beetles were classified as detritivores. *Nicrophorus quadripunctatus* and *N*. *concolor* are both carrion beetles of the family Silphidae, which feeds on vertebrate carcasses. Their ^137^Cs activity concentrations were similar to carnivore Carabidae beetles. On the other hand, the dung beetle, *Geotrupes laevistriatus*, and the giant weevil, *Sipalinus gigas*, showed high ^137^Cs activity concentrations. The larvae of these species and adult dung beetles feed on the dung of mammals, and adults are also attracted to decaying carrion and fungi. In study of radioactive contamination in insect species in Poland, Mietelski et al. [13,33] suggested the forest dung beetle as a suitable species for biomonitoring of radioactive contamination because it has high ^137^Cs concentrations compared to herbivores. The larvae of giant weevils feed on dead or decaying wood. It is possible that giant weevils have high levels ^137^Cs because decaying wood accumulates ^137^Cs because of the presence of wood-decaying fungi.

Among omnivorous insects, the camel cricket, *Diestrammena ssp*. had especially large CR values. This species is eats a wide variety of organic materials on the forest floor, including litter, fungi, and other invertebrate species. The CR values of detritivores and omnivores varied highly across sampling sites, likely indicating the nonuniform nature of ^137^Cs accumulation in fungi and decaying organic materials, as well as the varied diet of individual insect species [12,15].

### ^137^Cs transfer in the forest insect food web

In this study, litter and other forest components that were highly contaminated with ^137^Cs, such as fungi, decaying wood, bryophytes, and lichens were considered to be primary sources of ^137^Cs transfer into the forest insect community. Detritivores showed higher ^137^Cs accumulation than herbivores, confirming that uptake of ^137^Cs into insect ecosystems occurs through the detritus-based food chain and not through the plant-based food chain as previous studies have suggested [6,7].

With regard to ^137^Cs transfer through trophic levels, ^137^Cs activity concentrations of carnivorous insects were higher than those of herbivores but not higher than those of detritivores. Because carnivorous insects were represented by ground-dwelling beetles in this study, a significant proportion of their diet might have comprised detritivorous organisms. Therefore, this result might indicate a decrease in ^137^Cs activity concentrations in carnivores compared with that in detritivores. Rudge et al. [8] reported similar findings in a study of grassland invertebrate communities in the United Kingdom after the Chernobyl accident and suggested that ^137^Cs activity concentrations decrease up the food chain. Using stable carbon and nitrogen isotope ratio analysis of organisms in a terrestrial and stream ecosystem, Sakai et al. (2016) likewise observed dilution of ^137^Cs as it moved from lower to higher trophic levels [7]. These findings are the opposite of what has been observed regarding the bioaccumulation of ^137^C in fish species. In general, fish species at higher trophic levels will have higher activity concentrations of radiocesium than those farther down the food chain [34,35].

Our results do not provide clear evidence to support the idea of dilution of radiocesium as it moves up the food chain because we did not collect insect species that had a direct predator–prey relationship and because our measurements possibly overestimated the ^137^Cs activity concentrations in detritivores. We measured insect whole-body ^137^Cs activity concentrations similar to the reported insect ^137^Cs activity concentrations in previous studies because of the difficulty in collecting sufficient biomass for ^137^Cs measurements from dissected individual tissue types [8,12–17]. Thus, the high ^137^Cs concentrations measured in detritivorous insects may have been partly due to the presence of highly contaminated organic matter and soils in the digestive systems of sampled insects. Mietelski et al. (2003) found that measurement of ^137^Cs in dung beetles could be influenced by food remains in the digestive system. In addition, studies on the assimilation of radiocesium by earthworms have shown that little absorption occurs from contaminated gut contents [36], with radioactivity concentrations in earthworm tissues being far lower than those in the gut [8,37]. However, ^137^Cs activity concentrations of fish typically have been measured in dissected muscular tissues, so there was no contamination by gut contents. Therefore, the overestimation of ^137^Cs activity concentrations of whole-body samples should be taken into account when evaluating the ^137^Cs transfer through the detritus-based food chain and accumulation/dilution of ^137^Cs. Future studies that focus on^137^Cs activity concentrations in predator–prey relationships and on the bioavailability of soil-associated and litter-associated ^137^Cs for tissue incorporation will lead to better understanding of the transfer of ^137^Cs through the food web.

^137^Cs contaminations of arthropods are expected to gradually decrease as ^137^Cs decline activity concentrations in forest litter [3]. Because most herbivorous insect species have a reproductive cycle of 1 year or less, their ^137^Cs activity concentrations should reflect the level of contamination of their diet of the year in which they reproduced. In other functional feeding groups, members of some species may live for several years; for example, the life expectancy of Carabidae adults is 1 to 4 years. However, in the invertebrates that constitute their diet, the biological half-life of ^137^Cs is typically several days to a month [32]. Thus, the radiocesium concentration in insects of this species would also reflect the current contamination levels of the organisms that constitute their diet.

## Conclusions

Understanding the movement of ^137^Cs through ecosystems is essential for the management of radiation contamination and risk assessment in forest environments. This study investigated ^137^Cs transfer in forest insect communities in areas contaminated by the Fukushima Dai-ichi Nuclear Power Plant Accident. The results showed that ^137^Cs activity concentrations were lowest in herbivores and highest in carnivores, detritivores, and omnivores. The level of contamination in each of the four functional feeding groups of insects reflected the level of contamination of the materials and organisms that constitute their diets. Detritorivorous species had the highest levels of contamination, confirming findings of previous studies that these species play a significant role in ^137^Cs transfer into the forest ecosystem via consumption of highly contaminated forest litter. The nonuniform distribution of ^137^Cs in the forest environment is not only because of litter but also because of other forest components that may have high levels of ^137^Cs contamination, including fungi, decaying wood, bryophytes, and lichens. Insect species that have high CR values or that live in highly contaminated substrates, such as dung beetles, camel crickets, and lichen moths, would be appropriate species for monitoring radiocesium activity concentrations or for studies of radiation effects on wildlife.

## Supporting Information

S1 Table Details of samples of forest components. ^137^Cs values and counting errors are shown.
(XLSX)

S2 Table Details of samples of insect components. ^137^Cs values and counting errors are shown.
(XLSX)
